# Latent Profile Analysis of Symptom Severity in Systemic Lupus Erythematosus (SLE) Patients: A Cross‐Sectional Study

**DOI:** 10.1155/nrp/6973657

**Published:** 2026-05-23

**Authors:** Xingyu Yang, Shiyu Pu, Yuguang Shang, Xue Leng, Xiaomeng Wang, Shuyu Han, Chuangyi Zhang

**Affiliations:** ^1^ School of Nursing, Peking University, Beijing, 100191, China, pku.edu.cn; ^2^ Department of Nursing, Shengjing Hospital of China Medical University, Shenyang, 110004, Liaoning, China, cmu.edu.cn

**Keywords:** individualized interventions, influencing factors, latent profile analysis, symptom management, symptom severity, systemic lupus erythematosus

## Abstract

**Objectives:**

To explore the latent profiles of symptom severity among systemic lupus erythematosus (SLE) patients and analyze the influencing factors associated with these different latent profiles, providing clinical evidence for the management of SLE symptoms.

**Methods:**

Using convenience sampling, a questionnaire survey was conducted among SLE patients at Shengjing Hospital of China Medical University employing the SLE Symptom Checklist (SSC) between November 2022 and July 2023. Latent profile analysis (LPA) was performed to assess the severity of patients’ symptoms, and univariate analysis along with multinomial logistic regression was utilized to analyze the factors influencing the latent profiles.

**Results:**

A total of 420 patients were included in the study. The results of LPA revealed that the severity of symptoms among SLE patients could be categorized into three latent profiles: mild (75.4%), moderate (17.6%), and severe (6.9%). The multinomial logistic regression analysis showed that male participants were more likely to belong to the “Severe Symptom Type” (*p* = 0.046, OR = 2.86). Employed participants had a lower likelihood of being classified into the “Moderate Symptom Type” (*p* = 0.027, OR = 1.86) and “Severe Symptom Type” (*p* = 0.002, OR = 0.213). Unmarried participants were more likely to be classified into the “Moderate Symptom Type” (*p* = 0.027, OR = 1.86).

**Conclusion:**

Our study enhanced understanding of SLE’s complexity, identified gender, employment, and marital status as key factors, and advised targeted nursing for high‐risk patients. Clinicians should consider SLE’s system involvement and strengthen social support.

## 1. Introduction

Systemic lupus erythematosus (SLE) is a significant global health concern, widely affecting populations across the world [1]. SLE, a systemic autoimmune disorder, commonly affects the skin, kidneys, joints, and nervous system, causing diverse symptoms like rashes, nephritis, arthritis, and neuropsychiatric (NP) issues [2–7]. Meanwhile, SLE patients may experience a diverse range of adverse effects stemming from chronic comorbidities, opportunistic infections, and long‐term use of medications [8, 9]. The extensive range of effects and varied symptoms associated with SLE pose significant clinical challenges; therefore, the importance of effective symptom management in SLE is underscored.

In the realm of SLE symptom severity research, notable studies have emerged, such as the investigation conducted by Nyman et al. [10], which scrutinized data from an extensive cohort of 21,101 participants. This comprehensive analysis illuminated the general characteristics and the manifestation of disease severity of SLE patients. Dequattro et al. [11] conducted an analysis revealing racial disparities in SLE symptom severity, with SLE more severe among U.S. Asian patients compared to White patients, and Asian patients and non‐White groups with younger ages at diagnosis had greater organ damage than White patients. Moreover, several studies have underscored that some factors can influence the clinical manifestations of SLE. For example, Ambrose et al. [12] found that SLE patients of different ages exhibited varying clinical phenotypes, with arthritis being more prevalent in older patients, whereas renal disease, alopecia, and aphthous ulcerations were more common in younger individuals. The aforementioned studies scrutinized SLE symptom severity and clinical manifestations; however, they did not reveal the intricate linkage between the diverse influencing factors and SLE symptom severity, or their analysis was confined to a limited set of factors.

Latent profile analysis (LPA) stands as a cornerstone technique in individual‐centered research, enabling the elaboration of intricate models from proposed indicators to streamline the optimal model selection [13]. Widely adopted in studies, it categorizes samples according to distinct characteristics, fostering a granular individual‐level analysis [13]. Lately, LPA has been widely applied among diverse populations, highlighting its importance in understanding heterogeneous groups. For example, Băjenaru et al. [14] utilized LPA to uncover distinct latent profiles of quality of life (QoL) among senior adults. Zhong and Zeng [15] applied latent class mixture models (LCMM) in a longitudinal study to reveal significant associations between gynecological disorders and elevated insomnia/depression trajectories in middle‐aged women. LPA has also been applied extensively in studies pertaining to SLE. For example, Connor et al. [16] employed LPA and structural equation modeling to investigate the intricate relationships between the type of census tract inhabited by African American women with SLE, their subjective perceptions of community disorganization, and the manifestation of depressive symptoms within the Atlanta metropolitan region. In another study [17] involving Connor, LPA was utilized to delve into the association between sociodemographic characteristics of African American female SLE patients and the accrual of organ damage.

Acknowledging the significance of symptom management in SLE, there exists a notable gap in analyzing predictors of symptom severity and clinical manifestations. The LPA offers an individual‐centered approach to stratifying SLE symptom severity. Therefore, our study employed the LPA to delve into the distinguishing features of potential symptom severity categories among SLE patients and to elucidate the intricate relationships between various influencing factors and these categories. This endeavor aims to inform the development of tailored interventions for alleviating clinical symptoms in SLE patients.

## 2. Methods

### 2.1. Study Setting and Participants

A cross‐sectional study using convenience sampling was conducted between November 2022 and July 2023 in Shengjing Hospital of China Medical University. The inclusion criteria in this study were as follows: (1) participants diagnosed with SLE according to the 2019 EULAR‐ACR criteria [18]; (2) age ≥ 14 years; (3) clear consciousness, able to communicate and read normally; (4) voluntary participation in the study. Exclusion criteria included participants with severe lupus NP disorders causing cognitive impairment or other physical symptoms that prevented survey completion.

### 2.2. Ethical Considerations

This study was approved by the Ethics Committee of Shengjing Hospital, China Medical University (2023PS951K), and all subjects provided informed consent.

### 2.3. Measure

#### 2.3.1. Demographic and Clinical Variables

This survey was designed collaboratively by researchers after reviewing literature and consulting with nursing research experts. It includes demographic information (age, gender, ethnicity, primary caregiver, education level, marital status, and employment status) and disease‐related information (duration of SLE diagnosis, use of corticosteroids, use of immunosuppressive drugs, duration of medication, and presence of comorbidities).

#### 2.3.2. Symptom Checklist

We utilized the SLE Symptom Checklist (SSC), devised by Grootscholten et al. [19] in 2003, referencing Yan et al.’s application of its Chinese version [20], a 38‐item self‐assessment scale employing a Likert scale ranging from 0 to 3, to evaluate the symptoms and their severity among participants in the preceding month prior to their visit. Higher scores indicate more severe symptoms. The Cronbach’s α coefficient for this scale in this study was 0.923, indicating good reliability.

### 2.4. Sample Size Calculation

Regarding the LPA model, there is no universally acknowledged authoritative method for calculating sample size, and in principle, larger sample sizes are preferable. Our study has included a larger sample size compared to previous research [14].

### 2.5. Data Collection

Before the survey, researchers distributed paper questionnaires and QR codes for electronic questionnaires to two data collectors and provided training on standardized survey language. Participants chose between paper or electronic questionnaires and filled them out carefully. For participants unable to complete the questionnaire themselves, data collectors administered the questionnaire face‐to‐face. For outpatient participants who could not complete the questionnaire due to time constraints, data collectors obtained consent, collected phone contact information, scheduled follow‐ups, and completed the questionnaire via phone. Paper questionnaire data were entered into a database by two people for verification, and electronic questionnaires were collected through Wenjuanxing, with data downloaded from the website backend.

### 2.6. Data Analysis

Data analysis was performed using IBM SPSS 26.0 and Mplus 8.3. Two‐sided tests were used, with a significance level of *p* < 0.05. Descriptive statistics for normally distributed quantitative data were expressed as mean ± standard deviation, and categorical or ordinal data were described using frequencies and percentages. Mplus 8.3 software was used to explore LPA for detecting latent features of SLE symptom severity. The number of profiles was increased from 1 to 4 to determine the best‐fit indices. Model fitting was assessed using likelihood ratio tests and the following criteria: Akaike information criterion (AIC) and Bayesian information criterion (BIC), with smaller values indicating better model fit. An entropy value closer to 1 indicates more accurate classification. The bootstrap likelihood ratio test (BLRT) was used to compare model fit differences. Based on the LPA results, the best classification model for SLE symptom severity was determined. SPSS 26.0 was used for chi‐square tests, rank‐sum tests, or one‐way ANOVA to compare general information differences among participants with different symptom severity categories. Unordered multinomial logistic regression was used with latent categories of SLE symptom severity as the dependent variable, and factors with significant differences from univariate analysis as independent variables, to explore factors affecting different latent categories of SLE symptom severity.

## 3. Results

### 3.1. Demographic Characteristics

Table 1 depicts the results of descriptive analysis. The present study included 420 participants aged 14–78 years, with a mean age of 36.98 years (SD = 12.78), and 91% of them were female. The majority of the participants were of Han Chinese (86.4%), had comorbidities (87.1%), were married (67.9%), and were taking hormone medications (96.7%). Approximately half of the participants were employed (51%). Regarding education levels, the distribution was as follows: 24 individuals (5.7%) had primary school education, 104 (24.8%) had junior high school education, 89 (21.2%) had high school or vocational school education, 99 (23.6%) had college education, and 104 (24.8%) had undergraduate or higher education. In terms of primary caregivers, 344 individuals (81.9%) cared for themselves, 69 (16.4%) were cared for by family members, and 7 (1.7%) had other caregivers. The average duration of lupus diagnosis was 6.705 years (ranging from 0.1 to 40 years). The average duration of hormone medication use was 6.424 years (ranging from 0 to 40 years).

**Table 1:** Participant characteristics and between‐group comparisons of different potential categories (*N* = 420).

Characteristics	*N* (%), *M* ± SD	*χ* ^2^/*Z*/*F*	*p*
Sex			
Male	38 (9)	7.023^a^	0.030^∗^
Female	382 (91)		
Race			
Han	363 (86.4)	2.815^a^	0.245
Minority	57 (13.6)		
Marital status			
Married	285 (67.9)	8.365^a^	0.015^∗^
Unmarried	135 (32.1)		
Primary caregiver			
Myself	344 (81.9)	6.516^a^	0.164
Family members (spouse, kids, or other relatives)	69 (16.4)		
Otherwise	7 (1.7)		
Employment			
Employed	214 (51)	20.023^a^	< 0.01^∗∗^
Unemployed	206 (49)		
Comorbidities			
Yes	366 (87.1)	4.878^a^	0.087
No	54 (12.9)		
Duration of hormone medication	6.424 ± 5.6374	0.498^b^	0.608
Duration of diagnosed SLE	6.705 ± 5.7676	0.439^b^	0.645
Age	36.98 ± 12.78	2.434^b^	0.089
Education			
Primary school or less	24 (5.7)	6.367^c^	0.041^∗^
Junior school	104 (24.8)		
Senior high school	89 (21.2)		
College	99 (23.6)		
University or above	104 (24.8)		

^a^Chi‐square test.

^b^Analysis of variance.

^c^Wilcoxon rank‐sum test.

^∗^ *p* < 0.05.

^∗∗^ *p* < 0.01.

### 3.2. Incidence and Severity of Symptoms in Participants

As presented in Table 2, among 415 participants (98.88%), at least one symptom was reported within the past month, and there was an average of 10 symptoms per patient. The five most prevalent and severe symptoms identified were fatigue, hair loss, photosensitivity, memory impairment, and facial swelling.

**TABLE 2:** Symptom prevalence and severity of participants (*N* = 420).

Symptom	Positive cases *N* (%)	Severity score^a^	
		M (P25, P75)	Mean
Fatigue	276 (65.7)	1 (0, 1)	0.887
Hair loss	251 (59.8)	1 (0, 1)	0.867
Sensitivity to sunlight	239 (56.9)	1 (0, 1)	0.803
Disturbed memory	229 (54.5)	1 (0, 1)	0.738
Chubby cheeks/face	219 (52.1)	1 (0, 1)	0.718
Painful joints	195 (46.4)	0 (0, 1)	0.642
Skin rash	177 (42.1)	0 (0, 1)	0.613
Weight gain	178 (42.4)	0 (0, 1)	0.584
Mood changes	176 (41.9)	0 (0, 1)	0.572
Nightmares	158 (37.6)	0 (0, 1)	0.56
Muscle weakness	181 (43.1)	0 (0, 1)	0.555
Vulnerable skin	137 (32.6)	0 (0, 1)	0.505
Shortness of breath	141 (33.6)	0 (0, 1)	0.436
Blurred vision	143 (34)	0 (0, 1)	0.432
Loss of concentration	121 (28.8)	0 (0, 1)	0.391
Painful muscles	113 (26.9)	0 (0, 1)	0.359
Headache	100 (23.8)	0 (0, 0)	0.305
Stomach complaints	94 (22.4)	0 (0, 0)	0.289
Ulcers in mouth or throat	99 (23.6)	0 (0, 0)	0.284
Poor wound healing	92 (21.9)	0 (0, 0)	0.283
Facial hair growth	100 (23.8)	0 (0, 0)	0.281
Spontaneous bruises	86 (20.5)	0 (0, 0)	0.27
Sensitivity to artificial light	77 (18.3)	0 (0, 0)	0.257
Itch	83 (19.8)	0 (0, 0)	0.253
Ankle edema	74 (17.6)	0 (0, 0)	0.236
Muscle cramps	74 (17.6)	0 (0, 0)	0.23
More appetite	66 (15.7)	0 (0, 0)	0.218
Less appetite	52 (12.4)	0 (0, 0)	0.195
Blue/purple stretch marks on the skin	58 (13.8)	0 (0, 0)	0.195
“White” fingers in cold weather	60 (14.3)	0 (0, 0)	0.193
Nausea/vomiting	57 (13.6)	0 (0, 0)	0.18
Red and painful eyes	58 (13.8)	0 (0, 0)	0.171
Fainting	46 (11)	0 (0, 0)	0.135
Chest pain	50 (11.9)	0 (0, 0)	0.133
Pimples	36 (8.6)	0 (0, 0)	0.11
Pain while breathing	26 (6.2)	0 (0, 0)	0.074
Genital sores	9 (2.1)	0 (0, 0)	0.033
Fits	3 (0.7)	0 (0, 0)	0.007

^a^The scores do not follow a normal distribution and are represented as M (P25, P75), with the mean value provided for supplementary evaluation.

### 3.3. LPA of Symptom Severity in Participants

LPA was performed on the symptom severity of 420 participants. This analysis employed nine dimensions derived from 38 symptom items as indicators. Models with 1–4 latent profiles were sequentially fitted, starting from a baseline model with one profile. The fit indices for each model are shown in Table 3. Among the models with 1–4 profiles, the BLRT tests for the 2‐, 3‐, and 4‐profile models were significant (*p* < 0.05). As the number of profiles increased, both AIC and BIC values decreased, suggesting that models with more profiles might fit the data better. The entropy value of the 3‐profile model (0.95) was higher than that of the 4‐profile model (0.92). Additionally, the 3‐profile model (with profile proportions of 0.069/0.176/0.754) showed better profile heterogeneity and clinical interpretability compared to the 4‐profile model (with profile proportions of 0.026/0.111/0.171/0.690). Therefore, the 3‐profile model was selected as the optimal model for categorizing the severity of symptoms in participants.

**TABLE 3:** Comparison of fit indices between models (*N* = 420).

Number of profiles	Akaike (AIC)	Bayesian (BIC)	Entropy	BLRT test *p*	Marginal prevalence for latent profile
1	14,039.82	14,112.54	1	—	—
2	12,455.98	12,569.11	0.98	0.01	0.166/0.833
3	12,157.59	12,311.12	0.95	0.01	0.069/0.176/0.754
4	11,994.82	12,188.75	0.92	0.01	0.026/0.111/0.171/0.690

Abbreviation: BLRT, bootstrap likelihood ratio test.

**FIGURE 1:**
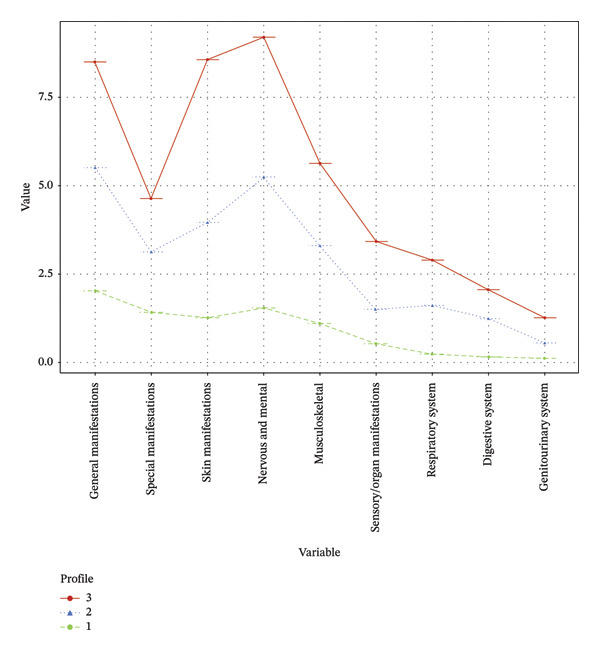
Distribution of characteristics across three potential profiles of symptom severity in participants.

Based on the LPA, participants were classified into three profiles: Profile 1 (75.4%, 317 participants), Profile 2 (17.6%, 74 participants), and Profile 3 (6.9%, 29 participants). The analysis of symptom severity across nine dimensions in Figure 1 identified three profiles: mild (Profile 1), moderate (Profile 2), and severe symptom types (Profile 3). All profiles had higher mean scores in “general manifestations,” “special manifestations,” “skin manifestations,” “nervous and mental,” and “musculoskeletal” dimensions and lower scores in “sensory organ manifestations,” “respiratory system,” “digestive system,” and “genitourinary.” Profile 1 exhibited three peaks, namely, “general manifestations,” “skin manifestations,” and “nervous and mental.” Profile 2 displayed two peaks, specifically “general manifestations” and “nervous and mental,” whereas Profile 3 demonstrated relatively consistent scores, forming a platform. We explored the symptom scores across nine dimensions for these three profiles, and significant differences were observed among all dimensions of the three profiles (*P* < 0.05) (see Table 4).

### 3.4. Univariate Analysis of Symptom Severity Latent Categories in Participants

The univariate analysis comparing demographic factors among different latent categories uncovered significant differences in gender, marital status, education level, and employment status across the three categories (*p* < 0.05), as shown in Table 1.

### 3.5. Multinomial Logistic Regression of Symptom Severity Latent Categories in Participants

Table 5 presents the results of multinomial logistic regression. Employing an unordered multinomial logistic regression model with symptom severity latent categories as the dependent variable (with “Mild Symptom Type” as the reference), the results showed that male participants were more likely to belong to the “Severe Symptom Type” (*p* = 0.046, OR = 2.86) compared to female participants, whereas no significant sex difference was observed in the “Moderate Symptom Type” (*p* > 0.05). Additionally, employed participants had a lower likelihood of being classified into the “Moderate Symptom Type” (*p* = 0.027, OR = 1.86) and the “Severe Symptom Type” (*p* = 0.002, OR = 0.213) compared to those who were unemployed. Furthermore, compared to married patients, those who were unmarried were more likely to be classified into the “Moderate Symptom Type” (*p* = 0.027, OR = 1.86). No statistically significant associations were found for other variables, including education level and duration of hormone use (*p* > 0.05).

**TABLE 4:** Multinomial logistic regression of potential symptom categories in participants.

Variables	Moderate symptom type		Severe symptom type	
	OR (95% CI)	*p*	OR (95% CI)	*p*
Sex (reference = female)	0.444 (0.129, 1.535)	0.2	2.86 (1.019, 8.029)	0.046^∗^
Employment (reference = unemployed)	0.533 (0.304, 0.934)	0.028^∗^	0.213 (0.081, 0.563)	0.002^∗∗^
Marital status (reference = married)	1.86 (1.074, 3.22)	0.027^∗^	1.87 (0.831, 4.209)	0.131
Duration of hormone use	0.977 (0.93, 1.026)	0.351	1.003 (0.94, 1.07)	0.931
Education	0.851 (0.68, 1.066)	0.161	0.933 (0.663, 1.313)	0.69

Abbreviation: OR, odds ratio.

^∗^ *p* < 0.05.

^∗∗^ *p* < 0.01.

**TABLE 5:** SSC value across three latent profiles.

Value	M ± SD			*F*	*p*
	Profile 1	Profile 2	Profile 3		
Total	8.26 ± 8.522	26.00 ± 5.557	46.14 ± 8.522	951.750	< 0.01
General manifestations	2.02 ± 1.395	5.51 ± 2.179	8.45 ± 2.229	309.361	< 0.01
Special manifestations	1.39 ± 1.172	3.15 ± 1.392	4.62 ± 1.916	126.351	< 0.01
Nervous and mental	1.53 ± 1.546	5.19 ± 2.621	9.28 ± 2.671	307.350	< 0.01
Skin manifestations	1.26 ± 1.518	3.59 ± 2.257	8.55 ± 2.910	261.607	< 0.01
Musculoskeletal	1.08 ± 1.178	3.35 ± 1.869	5.59 ± 2.910	170.385	< 0.01
Sensory organs manifestations	0.51 ± 0.171	1.50 ± 1.126	3.41 ± 1.524	168.678	< 0.01
Respiratory system	0.21 ± 0.469	1.59 ± 1.046	2.90 ± 1.291	289.954	< 0.01
Digestive system	0.15 ± 0.455	1.20 ± 1.249	2.10 ± 1.319	137.698	< 0.01
Genitourinary	0.11 ± 0.355	0.55 ± 0.830	1.24 ± 1.300	62.308	< 0.01

*Note:* Profile 1: mild (*n* = 317, 75.4%); Profile 2: moderate (*n* = 74, 17.6%); Profile 3: severe (*n* = 29, 6.9%).

## 4. Discussion

Our research categorizes SLE patients into mild, moderate, and severe profiles via LPA. Previous studies have also applied latent‐class cluster analysis (LCA) to examine symptom severity in SLE, such as the multinational study by Touma et al. [21], which characterized symptom‐based subgroups across the United States and five European countries. In contrast, our study focused on the Chinese SLE population; it aimed to characterize symptom severity profiles among Chinese SLE patients and explore associated influencing factors. We seek to provide a scientific foundation for identifying at‐risk patient groups and facilitating the development of tailored interventions for clinical implementation.

Our research revealed the multifaceted and intricate characteristics of SLE symptoms, encompassing a spectrum of clinical manifestations. These manifestations extend from mild conditions, exemplified by blue/purple stretch marks on the skin and pimples, to severe conditions, notably including painful joints and other forms of organ dysfunction. This observation aligns with the consensus derived from the existing literature, emphasizing the broad range and complexity of SLE’s clinical manifestations [6]. The diversity of symptoms of SLE mainly stems from its complex pathogenesis and extensive systemic involvement [22]. To address the diagnostic and therapeutic challenges posed by SLE’s diverse symptomology, it is suggested to adopt multifaceted approaches. Firstly, given the multifaceted influences on SLE symptoms, the diagnosis and treatment process ought to encompass a thorough consideration of the patient’s genetic predispositions, hormonal milieu, immune status, and environmental determinants, thereby facilitating the development of an individualized therapeutic protocol. Secondly, it is advised that clinical practice embraces strengthened multidisciplinary teamwork in order to guarantee patients access to holistic and efficacious treatment modalities. Thirdly, to deepen understanding, researchers should continue to investigate the diverse manifestations and underlying immunopathological mechanisms of the disease by examining various symptom profiles.

In the present study, SLE patients were categorized into three profiles, “mild symptom type,” “moderate symptom type,” and “severe symptom type,” based on model findings. By observing the severity of symptoms in 9 dimensions of participants in different profiles in Figure 1, we can also draw some conclusions. Firstly, it is worth noting that in the severe symptom type, the severity of “skin manifestation” is notably elevated, whereas in the moderate and mild types, its severity is not as prominent in comparison to other dimensions. This observation implies that the presence of pronounced skin manifestations in SLE patients may serve as an indicator of overall disease severity. Such an inference underscores the importance of promptly initiating multidimensional and multifaceted therapeutic interventions that address the comprehensive symptoms of SLE patients. This approach facilitates a more holistic amelioration of SLE disease progression. Additionally, it is apparent that both severe and moderate symptom types exhibit pronounced severity across the dimensions of “general manifestations” and “nervous and mental,” with the latter being a well‐established commonality in SLE, as corroborated by prior research endeavors. For instance, through the analysis of NP events in SLE patients, Hanly et al. [23] found that over half of the patients (52.1%) experienced one or more NP events, while more than a quarter of the patients (26.7%) experienced two or more. This finding provides guidance for optimizing the allocation of intervention resources in clinical settings, emphasizing the importance of paying closer attention to patients’ general manifestations, skin manifestations, and NP aspects to ensure maximal treatment efficacy.

The results of our study revealed distinct patterns of association between sex and symptom severity profiles in SLE patients. Specifically, relative to the mild symptom group, male SLE patients were significantly associated with an increased likelihood of belonging to the severe symptom type but not with membership in the moderate symptom type. Previous studies have consistently confirmed that, compared to female SLE patients, male SLE patients typically exhibit more severe manifestations of the disease. For instance, Santamaría‐Alza et al. [24] discovered that male SLE patients exhibit more severe disease manifestations and poorer prognoses across several key indicators: significantly elevated European Consensus Lupus Activity Measurement (ECLAM) scores, which are indicative of heightened disease activity, as well as prolonged hospitalization durations, higher readmission rates, and an increased risk of mortality. Previous studies have indicated that certain biological and behavioral factors, such as gender differences in sex hormone levels and ultraviolet (UV) exposure, may be associated with disease activity in SLE [25–28]. Our findings, from the perspective of patient‐reported symptom scores, provide evidence supporting the existence of clinical gender differences among SLE patients. Drawing upon the findings, we formulate the following recommendations: First, craft tailored nursing plans for male patients in clinical settings, focusing on early monitoring and timely intervention of relevant indicators to prevent high disease activity and systemic damage in male patients and effectively manage disease progression. Second, researchers are encouraged to delve deeper into gender‐specific disease mechanisms to establish a robust theoretical basis for the development of precise nursing protocols.

The findings of this study indicate a significant association between employment status and symptom severity profiles. Employment was associated with a lower likelihood of belonging to both the moderate symptom type and the severe symptom type compared to the mild symptom type. This finding aligns with the study by Touma et al. [21] presenting a positive correlation between work productivity impairment and symptom severity in SLE patients. Similar conclusions have been drawn in other disease research realms. For instance, by examining the relationship between employment status and disease severity in fibromyalgia patients, Mohabbat et al. [29] observed a significant association between employment status and symptom severity, with not working patients experiencing increased pain symptoms compared to those who are employed. On one hand, these findings indicate that SLE symptoms, such as painful joints and disturbed memory, may significantly impair patients’ ability to participate in work and social functioning. On the other hand, employment status is often associated with relatively stable financial income and social support networks, which have been reported in multiple studies to correlate with health outcomes in patients [30–32]. For instance, stable income may alleviate financial pressure related to healthcare [31], while the workplace environment can provide supportive resources such as social interaction, identity affirmation, and institutional safeguards [32]. These factors may collectively correlate with reduced self‐reported symptom severity in SLE patients. Given the outcomes observed, we recommend that clinical practice incorporate assessments of employment status in SLE patients as a reference for developing individualized support plans. For patients who are unemployed or have unstable employment status, it is recommended to strengthen regular follow‐up and symptom assessments to facilitate timely clinical intervention and support, thereby promoting overall health and QoL.

The results of this study revealed that, compared with the mild symptom group, unmarried SLE patients were more likely to be classified into the moderate symptom group, indicating that unmarried patients exhibited more severe symptoms. This finding aligns with previous research regarding the association between marital status and symptom severity in chronic diseases. For example, Hosny et al. [33] found that among patients with multiple sclerosis (MS) relapses, single patients tended to experience more severe relapses, whereas those with stable marital status showed lower disease severity. Previous studies have suggested that marital status serves as an important source of social support and may be related to protective effects on disease management through psychological stress relief and enhanced self‐efficacy in disease self‐management [34–36]. Additionally, marital status may also be associated with health behavior adherence, as reminders and supervision from spouses may promote patient adherence to treatment plans [37–39]. Clinical practice should pay greater attention to unmarried SLE patients regarding psychosocial support and treatment adherence to help manage symptoms.

Several limitations to our study should be noted. First, the design of our cross‐sectional study inherently restricts our ability to evaluate the temporal stability of latent profiles, thereby underscoring the need for future longitudinal investigations to delineate the dynamic trajectory of symptom severity over time. Second, important clinical covariates that are directly related to severity in SLE, such as a disease activity score, organ involvement, and a damage index, are of value for the regression analysis. We agree that future studies could benefit from including more clinical covariates for a more thorough analysis. Third, the relatively small number of participants in the mild symptom profile may affect the stability of the latent profile model and the reliability of the multinomial logistic regression estimates involving this subgroup.

## 5. Conclusion

This study employed LPA to conduct an in‐depth analysis of the SLE patients, categorizing them into three profiles based on the severity of their symptoms. This deepened our comprehension of SLE’s complexity and heterogeneity. It also identified gender, employment, and marital status as key factors affecting symptom severity, paving the way for targeted nursing strategies for high‐risk patients. Our research advises clinicians to consider specific system involvement of SLE patients and strengthen social support networks to enhance overall patient health. Ultimately, our findings offer scientific and comprehensive guidance for SLE nursing practices.

## Funding

The authors received no specific funding for this work.

## Conflicts of Interest

The authors declare no conflicts of interest.

## Data Availability

The data that support the findings of this study are available from the corresponding author upon reasonable request.
